# Understanding intergroup violence justification: the role of ethnicity and perceived threat in Israeli society

**DOI:** 10.3389/fpsyg.2025.1508324

**Published:** 2025-02-18

**Authors:** Nir Rozmann

**Affiliations:** Western Galilee College, Acre, Israel

**Keywords:** perceived threat, intergroup violence, ethnicity, integrated threat theory, Israel

## Abstract

Research has shown that perceived realistic and symbolic threats are linked with negative attitudes and prejudice toward out-group members. Additionally, levels of perceived group threat regarding out-groups can affect intergroup violence justification. Based on the Integrated Threat Theory (ITT), the current study aimed to expand existing knowledge by examining a conceptual model in which perceived threat mediates the relationship between ethnicity and intergroup violence justification among Jews and Arabs in Israel. The study involved 324 Israeli-Jewish and 325 Israeli-Arabs, who answered questions regarding perceived out-group threat and intergroup violence justification. Findings revealed that (a) Jews were more likely to justify intergroup violence than Arabs, and (b) perceived realistic threat mediates the relationship between ethnic affiliation and intergroup violence justification only among Jews. These results underscore the importance of understanding intergroup conflicts in the field of criminology.

## Introduction

Heterogeneous societies often experience tensions and struggles over political power and resources, which can lead to negative attitudes, stereotypes, prejudice, and discrimination (Bahns, 2017; Esses, 2021; Quillian et al., 2019; Wirtz et al., 2016). Integrated Threat Theory (ITT) offers a comprehensive framework for understanding the roots of prejudice and intergroup conflict (Stephan et al., 1998, 2000). ITT is grounded in the idea that intergroup relations are influenced by the perceived threats that groups pose to each other.

Stephan et al. (1998) delineates two primary types of threats: Realistic threats and symbolic threats. Perceived realistic threat refers to the apprehension individuals feel regarding the tangible, material threats that out-groups may pose to their own group’s well-being, resources, or security. This includes fears related to competition for jobs, social services, or even physical safety (Hellmann et al., 2022; Stephan et al., 2000). In contrast, perceived symbolic threat pertains to the fear that out-group members may challenge the in-group’s values, norms, and identity. This type of threat often involves concerns about cultural dilution or the erosion of social cohesion (Davidov et al., 2020; Stephan et al., 1999; Wirtz et al., 2016).

Symbolic and realistic threats are crucial concepts in understanding intergroup relations and prejudice formation, as they reveal the underlying mechanisms that drive negative attitudes toward outgroups by highlighting how perceived threats to cultural values and tangible resources shape intergroup dynamics (Martínez et al., 2022; Nir and Sophie, 2018; Riek et al., 2006). These threats may elicit different outcomes due to their distinct nature and impact on individuals and groups. Research has shown that symbolic threats are more strongly associated with hate and tend to persist over time, forming the “solid core of prejudice,” whereas realistic threats are more likely to evoke anger and may be more transient (Stephan et al., 1998). Additionally, the salience of each type of threat can vary depending on the context and the specific outgroup involved. For example, economically powerful outgroups might elicit realistic threats, while socially marginalized groups may engender symbolic threats (Velasco González et al., 2008).

ITT provides a framework for understanding how perceived threats from ethnic out-groups can lead to prejudice and negative attitudes (Bahns, 2017; Chiricos et al., 2020; Nir and Sophie, 2018; Wirtz et al., 2016). In the context of ethnicity, ITT posits that members of a dominant ethnic group may experience symbolic and/or realistic threats, which can arise from cultural differences, competition for resources, or historical conflicts between ethnic groups (Halperin, 2008; Hirschberger et al., 2016). As a result, dominant ethnic groups may perceive immigrant groups as threatening their economic well-being or cultural identity, leading to negative attitudes and resistance to integration efforts (Chiricos et al., 2020; Davidov et al., 2020; Nir and Sophie, 2018). For instance, research has explored how symbolic and realistic threats manifest in Jewish perceptions of Arabs as the “other,” with Jewish as the ingroup experiencing various anxieties toward Arabs as the outgroup (Hirschberger et al., 2016; Rozmann and Yehuda, 2023a).

Other studies have consistently demonstrated a relationship between perceived realistic threats and negative attitudes (Appel et al., 2015; Canetti-Nisim et al., 2008; Martínez et al., 2022; Nir and Sophie, 2018). Studies have shown that individuals who perceive economic competition from immigrants tend to express more anti-immigrant sentiments (Appel et al., 2015; Croucher, 2013; Curşeu et al., 2007). These perceptions are often exacerbated during economic downturns, where the competition for resources becomes more pronounced. Similarly, perceived symbolic threats have been linked to increased prejudice, as individuals who felt their cultural identity was threatened by immigration were more likely to endorse negative stereotypes about immigrants (Davidov et al., 2020; Rios et al., 2018). A meta-analysis of 70 studies revealed that perceptions of refugees as both symbolic and realistic threats were the strongest correlates of negative attitudes (Cowling et al., 2019). Additionally, opposition to immigration in European countries was found to be associated with symbolic threat, specifically the fear of losing cultural and national identity (Croucher, 2013; Wirtz et al., 2016).

### Perceived threat and justifying intergroup violence

Research on ITT primarily focuses on its consequences for intergroup relations, demonstrating that perceived threat plays a crucial role in fostering prejudice, discrimination, and social control (Chiricos et al., 2020; Nir and Sophie, 2018; Riek et al., 2006; Rios et al., 2018). However, there has been limited attention to the potential consequences of group threat in justifying intergroup violence. Perceived threat may predict violence between social groups, as it can motivate psychological processes that enable individuals to overcome their aversion to violence and participate in intergroup conflict (Lantos and Molenberghs, 2021).

In the current study, I examine the role of perceived threat in justifying intergroup violence in Israel. The ongoing conflict between Jews and Arabs in Israel is a prolonged and violent struggle that has incurred significant human and material costs (Ayer et al., 2017; Canetti-Nisim et al., 2009; Cohen-Louck and Saka, 2017; Loewenthal et al., 2023).

The Israeli-Arab conflict is a complex and long-standing dispute over territory and self-determination in the region of former Mandatory Palestine. Its roots can be traced back to the late 19th century, but it intensified significantly in the mid-20th century. The conflict escalated after the 1947 United Nations Partition Plan, which proposed dividing the land into Jewish and Arab states (see Haushofer et al., 2010). Israel’s declaration of independence in 1948 led to the first Arab-Israeli War, resulting in Israel’s establishment but also the displacement of hundreds of thousands of Arabs. Since then, the conflict has been marked by several wars, territorial disputes, and failed peace attempts (Ayer et al., 2017; Halperin, 2008). Key issues include the Israeli occupation of the West Bank and Gaza Strip, the status of Jerusalem, Israeli settlements, borders, security concerns, and the Palestinian right of return. As such, the conflict has had far-reaching consequences, including multiple wars, ongoing violence, and significant humanitarian crises (Halperin and Bar-Tal, 2011; Halperin and Gross, 2011).

The socio-political context of the Israeli-Palestinian conflict further complicates perceptions of threat and justifications for violence. The prolonged nature of the conflict has led to a cycle of fear and aggression, where each side views the other as a perpetual threat, contributing to negative and hostile attitudes toward the out-group (Canetti-Nisim et al., 2008; Halperin, 2008; Nir and Sophie, 2018; Rozmann, 2024; Rozmann and Yehuda, 2023a). Israeli-Jews may often engage in “competitive victimhood” (CV), a phenomenon where individuals perceive their own group as having suffered more than the outgroup, particularly in the context of prolonged conflicts (Sharvit and Kremer-Sharon, 2023; Young and Sullivan, 2016). This CV can function as a psychological mechanism to justify violent actions against Palestinians, especially under conditions of high realistic threat. Bar-Tal (2007) found that heightened perceptions of threat from Palestinian groups correlate with increased support for military actions and violence against them (Bar-Tal, 2007). Similarly, Halperin and Gross (2011) demonstrated that when Israeli Jews perceive a higher threat from Palestinians, as they are more likely to endorse violent measures against them (Halperin and Gross, 2011). This phenomenon is often exacerbated during escalations of conflict, where political rhetoric amplify perceptions of threat (see Sela-Shayovitz, 2009). Rozmann and Yehuda (2023a) found a significant relationship between perceived realistic threat and intergroup violence justification among Israeli-Jewish in Israel. However, no relationship was found between perceived threat and intergroup violence justification within Israeli-Arabs (Rozmann and Yehuda, 2023b).

Given the importance of ITT in shaping attitudes toward outgroup members, this article explicitly focuses on the potential correlation between perceived threat and the justification of intergroup violence. Based on previous literature, I argue that group threat should be considered as a negative intergroup stressor. Therefore, I hypothesize that:

*H1*: Ethnic affiliation would be associated with intergroup violence justification, as Israeli-Jewish will justify intergroup violence more than Israeli-Arab participants.

*H2*: Perceived threat will mediate the link between ethnicity and intergroup violence justification.

### Overview of the current study

Previous studies suggest that perceived threat is linked to prejudice, discrimination, and negative attitudes toward outgroup members (Bahns, 2017; Chiricos et al., 2020). However, it remains unclear how perceived threat relates to the justification of intergroup violence. This article aims to fill this gap by examining the link between perceived threat and violence justification in the Israeli context. The ongoing conflict between Jews and Arabs in Israel is violent and has led to significant social categorization issues, resulting in negative attitudes from both sides (Halperin, 2008; Halperin and Gross, 2011; Rozmann and Nahari, 2021). While both groups, Jews and Israeli Arabs, are involved in ethnic violence that causes injuries and damage (Hitman, 2023; Shalhoub-Kevorkian and David, 2016), it is crucial to explore whether perceived threat correlates with the justification of such violence. Gaining preliminary insights in this area could guide further research and contribute to the development of policies and programs aimed at reducing feelings of threat and, consequently, violent attitudes.

## Method

### Participants

This study involved 659 respondents from Israel, who voluntarily participated in the research (for demographic characteristics see Supplementary Table S1).

### Measures

#### Intergroup violence justification

Participants responded to one statement with the prompt: “Please indicate whether you think “violence against out-group members (Israeli-Jewish or Israeli-Arab) can always be justified, never be justified, or falls somewhere in between. Response options ranged from 1 (never justifiable) to 10 (always justifiable; see Park et al., 2023, 2024).

#### Perceived realistic threat

To measure realistic threat, a modified version of the realistic threat scale was used (Stephan et al., 1998). This measure comprises nine items that address various threats, such as crime, job loss, and the economic costs of health, education, and welfare. Examples of items include, “Israeli-Arabs\Jewish increase the level of violence” and “Israeli-Arabs\Jewish endanger the health of Jews.” Participants responded using a 5-point Likert scale, ranging from 1 (strongly disagree) to 5 (strongly agree). The total score was calculated as the mean of the items. The scale demonstrated high internal consistency with a Cronbach’s alpha of 0.88.

#### Perceived symbolic threat

To assess the threats posed by perceived differences in values and beliefs between ingroup and outgroup members, eight items were used (Stephan et al., 1998). These items were rated on a 7-point Likert scale, ranging from 1 (not likely at all) to 7 (very likely). Examples of the items include, “The values and beliefs of Israeli-Arabs\Jewish are fundamentally different from those of most Jews\Arabs” and “Israeli-Arabs\Jewish value power more than Jews\Arabs.” The total score was calculated as the mean of the items. This measure had a Cronbach’s alpha of 0.82, indicating good internal consistency.

### Procedure

#### Data collection

This study was approved by Western Galilee College’s ethics committee (WGC-1219). Data was collected through an online survey administered via Google Drive from January 2024 to May 2024. Participants were recruited through social media postings on Facebook and WhatsApp, which invited individuals to participate in a study on responses to crime. All participants spoke Hebrew fluently. Participants received no compensation for completing the questionnaires. To minimize potential biases, I simplified the language of the questionnaire, keeping it short and free from professional jargon and complex terms.

After providing electronic informed consent, participants were directed to complete the online questionnaire. The questionnaire assured participants of anonymity and confidentiality, stated that they could skip any questions that made them uncomfortable, and informed them that they could withdraw from the survey at any time. Participants were also assured that their responses would be used solely for research purposes.

#### Data analysis

Following the initial correlation analysis, I employed Hayes’s SPSS PROCESS procedure (Hayes, 2017), specifically a parallel mediation analysis- Model 4- to investigate whether symbolic threat and\or realistic threat mediates the relationship between ethnicity and intergroup violence justification. This procedure estimates the significance of the indirect effect using a bootstrap approach, a non-parametric method that relies on repeated random resampling with replacement, yielding 95% bootstrapped confidence intervals (CIs) for the indirect effect.

## Results

Overall, the level of symbolic threat (*Mean* = 5.59, *SD* = 1.48, *Range* = 1–7) and the level of realistic threat (*Mean* = 3.46*, SD* = 1.18, *Range* = 1–5) were relatively high. T-test results indicated ethnic differences in perceived realistic threat, as Israeli-Arab were more likely to report high levels of realistic threat (*M* = 3.58, *SD* = 1.22) than Israeli-Jewish (*M* = 3.34, *SD* = 1.13), [*t* (657) = 2.67, *p* = 0.004]. No differences were found between Israeli-Jewish and Israeli-Arab in perceived symbolic threat (*t* < 1, *p* > 0.05). Israeli-Jewish were more likely to legitimize intergroup violence (*M* = 6.66, *SD* = 2.40) than do Israeli-Arabs (*M* = 6.15, *SD* = 2.34), [*t* (657) = 2.75, *p* = 0.003]. Several *t*-tests on religiosity, gender, and education were also conducted (see Supplementary Table S1). Also, Intergroup violence justification was positively correlated with both realistic and symbolic threats. As expected, the symbolic threat was positively related to realistic threat (see Supplementary Table S2).

**Figure 1:**
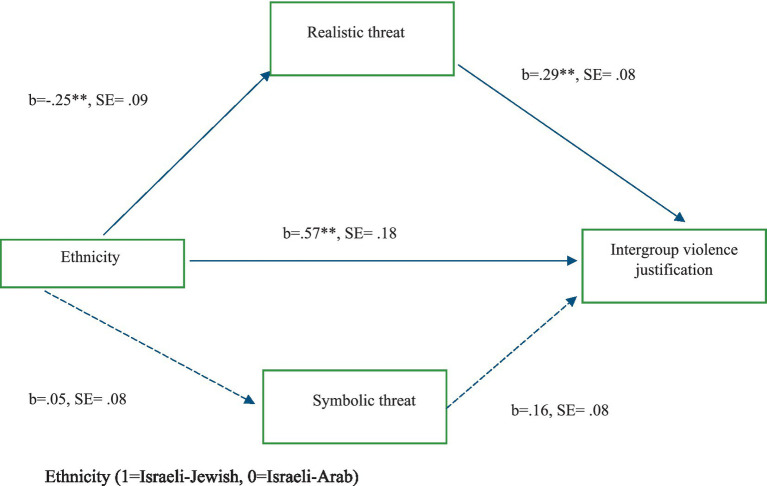
Path diagram for the association between ethnicity and intergroup violence justification mediated by perceived symbolic and realistic threat (*N* = 659). *p < 0.05; **p < 0.01.

In this study, I examined how perceived symbolic and realistic threats act as mediators in the relationship between ethnicity and intergroup violence justification. The overall PROCESS model, as can be seen in Figure 1, was statistically significant, *F* (3,655) = 10.41, *p* < 0.001. The total effect of ethnicity on violence was significant (*b* = 0.57, *SE* = 0.18, *p* = 0.006, 95% *CI* [0.15, 0.87]), indicating that ethnicity was associated with higher levels of intergroup violence justification. The direct effect remained significant when controlling for the mediators- perceived realistic and symbolic threat (*b* = 0.57, *SE* = 0.18, *p* = 0.002, 95% *CI* [0.21, 0.93]).The analysis of the first mediator (symbolic threat) revealed that ethnicity did not significantly predict symbolic threat (*b* = 0.05, *SE* = 0.09, *p* = 0.592), and symbolic threat showed a marginally significant relationship with violence (*b* = 0.16, *SE* = 0.08, *p* = 0.065). The indirect effect through symbolic threat was not significant (*b* = 0.008, *SE* = 0.02, 95% *CI* [−0.02, 0.04]). For the second mediator (realistic threat), ethnicity significantly predicted realistic threat (*b* = −0.25, *SE* = 0.09, *p* = 0.008), and realistic threat significantly predicted violence (*b* = 0.29, *SE* = 0.08, *p* < 0.001). The indirect effect through realistic threat was significant (*b* = −0.07, *SE* = 0.04, 95% *CI* [−0.15, −0.01]). These results suggest that while both direct and indirect effects are present, realistic threat serves as a significant mediator in the relationship between ethnicity and intergroup violence justification, while symbolic threat does not demonstrate significant mediation effects.

## Discussion

This preliminary explorative study builds on previous research regarding the relationship between threat perception and the justification of intergroup violence in Israel. Israel serves as a significant context for examining this issue due to the ongoing conflict between Jews and Arabs, which has fostered intolerance, prejudice, and hostility toward outgroup members (Ayer et al., 2017; Bar-Tal and Hammack, 2012; Halperin and Bar-Tal, 2011).

This study advances our understanding of the relationship between ethnicity and intergroup violence justification by testing a theoretical model in which perceived symbolic and realistic threat acts as mediators. The results revealed that (a) ethnicity significantly predicted intergroup violence justification, as Israeli-Jewish were more likely to legitimize intergroup violence then Israeli-Arab participants; (b) perceived realistic threat mediated the relationship between ethnicity and intergroup violence justification.

The results show that Israeli-Jewish tend to justify intergroup violence, then did Israeli-Arab participants. Previous research has shown a link between threat perception and justification of intergroup violence in Israel (see Rozmann and Yehuda, 2023a). Presumably, Israeli Jews may experience higher levels of perceived threat due to historical trauma, ongoing conflict, and security concerns, which could contribute to a greater willingness to justify violence as a means of self-protection (Ayer et al., 2017; Halperin and Bar-Tal, 2011; Halperin and Gross, 2011).

The second hypothesis regarding the mediating role of perceived threat on the link between ethnicity and intergroup violence justification was partially confirmed. While previous studies have shown that symbolic threat is more significant than realistic threat in predicting negative attitudes toward outgroup members (see Riek et al., 2006), the results suggest that only perceived realistic threat predicts intergroup violence justification. Realistic threats typically stem from the perception of competition over scarce resources, such as jobs, land, and political or economic power, as well as from threats to physical safety and the overall well-being of the ingroup (Rios et al., 2018; Stephan et al., 1998). In the context of the Israeli-Arab conflict, realistic threats likely include concerns about safety and security, which are tangible and immediate (see Halabi et al., 2021). This aligns with the finding that realistic threat predicts violence justification, as it may be seen as a direct response to perceived physical dangers (Noor et al., 2012). However, the absence of the relationship of symbolic threat with intergroup violence justification in the Israeli context may indicate that immediate security concerns overshadow cultural or ideological differences in driving violence justification. As such, in prolonged, violent conflicts like the Israeli-Arab situation, realistic threats may become more salient and influential in shaping attitudes toward intergroup violence.

In this point, it is essential to note that this study took place during the “Iron Swords” War between Israel and Hamas, which was launched in response to the country’s worst terror incident on October 7th, 2023. The brutal terrorist attack unfolded under horrific circumstances, including shootings, arson, the pursuit of those attempting to flee, and acts of severe sexual violence. As a result, many Israelis grapple with a complex mix of post-traumatic stress and profound grief (Feingold et al., 2024; Hasson-Ohayon and Horesh, 2024; Levi-Belz et al., 2024), which may be related with justifying aggressive responses, as violence justification.

## Limitations

This preliminary explorative study has several limitations. First, a non-probabilistic sampling method within the Israeli population was used. This approach limits the study’s external validity, as the sample was neither random nor representing of Israeli society. Future research should include a random and representative sample. Future research should also consider other factors that may influence intergroup attitudes, such as social identification, fear of terrorism, and preferences for social control. In addition, data collection was conducted online. Online surveys research has limitations including potential sampling bias and reduced representativeness due to unequal internet access. They also face challenges with data reliability, as it’s difficult to verify respondent identities and prevent multiple submissions, potentially compromising the validity of research findings. Relationships between ethnic groups (majority and minority) can vary significantly across different cultural contexts. Therefore, this study should be replicated in other sociopolitical settings.

Importantly, it is essential to note that this study was conducted during the “Iron Swords” war and the brutal attack on Israel on October 7^th^ 2023, which may explain way participants were particularly concerned about the realistic threat at the time the study was conducted.

## Implications and conclusion

This preliminary explorative study enhances both theoretical and practical understanding of ITT by examining the real-world consequences of perceived threats. Social psychological literature emphasizes the role of symbolic threats over realistic threats in shaping attitudes toward outgroup members. This study contributes to the empirical body of research by demonstrating that realistic threats, compared to symbolic threats, correlate with the justification of intergroup violence among Jews in Israel. This finding is especially relevant today, given the stressful environment in Israel during the ongoing ‘Iron Swords’ War, which influence Israeli’s public health (see Dahan et al., 2024).

The finding that perceived realistic threat mediates the relationship between ethnicity and the justification of intergroup violence in Israel carries significant implications for both policy and community relations. This insight suggests that addressing the underlying perceptions of threat—particularly those grounded in economic competition, security concerns, and resource scarcity—can be pivotal in mitigating intergroup tensions. Policymakers may need to develop targeted interventions that foster understanding and collaboration between different ethnic groups, thereby reducing the perception of threat and, consequently, the justification of violence. Additionally, community programs that promote dialogue and integration can help shift focus away from perceived threats, fostering a more cohesive society. Ultimately, recognizing the role of perceived realistic threats can guide efforts to create a more peaceful coexistence among diverse ethnic communities in Israel, and across the globe.

## Data Availability

The raw data supporting the conclusions of this article will be made available by the authors, without undue reservation.
