# Supplementary material for: Understanding intergroup violence justification: the role of ethnicity and perceived threat in Israeli society
Source: Front Psychol. 2025 Feb 18;16:1508324. doi: 10.3389/fpsyg.2025.1508324 (PMC11877392; doi:10.3389/fpsyg.2025.1508324)
Supplement: Supplementary file 1 [file Table_1.docx]

**Table S1**: *Demographic characteristics of the participants by ethnicity (N=659)*

| Characteristic | Total | | Jewish (n=324, 49.2%) | | | Arab (n=335, 50.8%) | | | Differences | |
| --- | --- | --- | --- | --- | --- | --- | --- | --- | --- | --- |
|  |  |  |  |  |  | |  |  | |  |
| Age (Years) |  |  |  |  |  | |  |  | |  |
| M (SD) (range: 19-70) | 32.29 (10.19) |  | 32.45 (9.96) |  | 32.15 (10.42) | |  | t(657)=.37 (p>0.05) | |  |
| Marital status n(%) |  |  |  |  |  | |  |  | |  |
| Single | 326 (49.5) |  | 145 (44.8) |  | 188 (56.1) | |  | χ2(1)=8.15 (p<.005) | |  |
| Married/partnered | 333 (50.5) |  | 179 (55.2) |  | 147 (43.9) | |  |  |  |  |
| Educational level n(%) |  |  |  |  |  | |  |  | |  |
| High school | 307 (46.6) |  | 153 (47.2) |  | 154 (46) | |  | χ2(1)=0.104 (p=.74) | |  |
| University or postgraduate degree | 352 (53.4) |  | 171 (52.8) |  | 181 (54) | |  |  |  |  |
| Religiosity n(%) |  |  |  |  |  | |  |  | |  |
| Secular | 342 (51.9) |  | 167 (51.5) |  | 175 (52.2) | |  | χ2(1)=0.032 (p=.85) | |  |
| Partly religious\religious | 317 (48.91) |  | 157 (48.5) |  | 160 (47.8) | |  |  |  |  |

**Table S2** *Correlations between the study variables (N=659)*

| Variable | *M* | *SD* | 1 | 2 | 3 | 4 |
| --- | --- | --- | --- | --- | --- | --- |
|  |  |  |  |  |  |  |
| 1. Age | 32.29 | 10.19 | - |  |  |  |
| 2. Intergroup violence justification | 6.4 | 2.38 | -.039 | - |  |  |
| 3. Realistic threat | 3.46 | 1.18 | -.156** | .158** | - |  |
| 4. Symbolic threat | 5.59 | 1.14 | -.061 | .126** | .327** | - |

^*^*p* < .05. ^**^*p* < .01.
